# Key glycometabolism during oocyte maturation and early embryonic development

**DOI:** 10.1530/REP-24-0275

**Published:** 2025-02-04

**Authors:** Yichuan Zhang, Tianjie Li, Yibo Wang, Yang Yu

**Affiliations:** ^1^ State Key Laboratory of Female Fertility Promotion, Center for Reproductive Medicine, Department of Obstetrics and Gynecology, Peking University Third Hospital, Beijing, China; ^2^ Key Laboratory of Assisted Reproduction, Peking University, Ministry of Education, Beijing Key Laboratory of Reproductive Endocrinology and Assisted Reproductive Technology, Beijing, China; ^3^ Clinical Stem Cell Research Center, Peking University Third Hospital, Beijing, China; ^4^ Beijing Key Laboratory of Reproductive Endocrinology and Assisted Reproductive Technology, Beijing, China; ^5^ Beijing Friendship Hospital, Capital Medical University, Beijing, China; ^6^ The Third Affiliated Hospital, Guangzhou Medical University, Institute of Obstetrics and Gynecology, Guangzhou, China

**Keywords:** glycometabolism, embryo development, oocyte maturation, zygote genome activation, blastocyst differentiation

## Abstract

**In brief:**

Glucose metabolism is central to the successful progression from oocyte maturation to blastocyst formation, influencing not only energy production but also key developmental decisions and epigenetic regulation. This review uncovers the intricate interplay between glucose, fatty acids, amino acids and nucleotides, highlighting their crucial roles in early embryonic development and exploring how noninvasive metabolic monitoring can revolutionize embryo selection and personalized assisted reproductive technologies, offering new hope for fertility treatments.

**Abstract:**

In recent decades, it has become increasingly clear that mammalian gametes and early embryos are highly sensitive to metabolic substrates. With advances in single-cell sequencing, metabolomics and bioinformatics, we now recognize that metabolic pathways not only meet cellular energy demands but also play a critical role in cell proliferation, differentiation and fate determination. Investigating metabolic processes during oocyte maturation and early embryonic development is thus essential to advancing reproductive medicine and embryology. This review highlights the intricate metabolic pathways, particularly glucose metabolism, that drive the transition from an oocyte to an embryo. These processes involve a complex interaction of signaling pathways, nutrient availability and environmental factors, with glucose metabolism not only providing essential energy but also offering a variety of metabolic substrates and intermediates that regulate developmental events, influence cell signaling and impact epigenetic modifications. This article emphasizes that future research will focus on regulating maternal metabolic environments and noninvasive metabolic monitoring of embryonic systems, particularly glucose metabolism, with promising opportunities to improve embryo selection and personalized assisted reproductive technologies, ultimately enhancing fertility treatment outcomes.

## Introduction

The quest to unravel the mysteries of life begins at the most fundamental biological level – understanding the metabolic orchestration that governs oocyte maturation and early embryonic development. In the dynamic and ever-evolving field of reproductive medicine and embryology, the study of metabolic processes is a critical endeavor that holds the key to unlocking the secrets of life’s inception. This review aims to shed light on the intricate metabolic pathways that are instrumental in the transition from an oocyte to an embryo, with a particular emphasis on glucose metabolism and its pivotal role in this delicate process (Gardner & Wale 2013, Gu *et al.* 2015).

The journey of an oocyte from maturation to fertilization and subsequent embryonic development is a marvel of biological engineering, where each step is meticulously regulated by metabolic cues (Mohebi & Ghafouri-Fard 2019, Sfakianoudis *et al.* 2021, Fei & Zhou 2022, Solovova & Chernykh 2022, Sahin *et al.* 2023, Wei *et al.* 2024). Metabolism during these early stages is not just about energy production; it is a complex interplay of signaling pathways, substrate availability and environmental influences that collectively ensure the successful progression of life (Gardner & Leese 1986, Hardy *et al.* 1989). As researchers delve deeper into the roles of glycolipid metabolism, glucose and its derivatives have emerged as vital elements of early life (Zou *et al.* 2022).

Oocyte maturation and early embryonic development processes are key to women’s reproductive success, and these complex events are closely related to glucose metabolism. First, the quality and developmental potential of oocytes are the basis for maintaining subsequent normal fertilization and early embryo development (Schultz *et al.* 2018). Maternal metabolic abnormalities can specifically lead to increased apoptosis of granulosa cells in preovulatory follicles, decreased oocyte size and meiotic maturation (Jungheim *et al.* 2010), decreased oocyte quality after ovulation (Minge *et al.* 2008) and reduced blastocyst survival rate and abnormal embryonic cell differentiation after fertilization (Minge *et al.* 2008). At the same time, maternal metabolic syndromes, such as obesity, diabetes and polycystic ovary syndrome (PCOS), cause abnormal development of the next generation by affecting the quality of oocytes (Huypens *et al.* 2016, Risal *et al.* 2019). Second, during early cleavage stages, glycolysis as an energy source for embryonic development is also crucial. It has been reported that *SLC2A3* and *LDHA*, which encode glycolytic enzymes, are continuously reduced in the stalled zygote to the eight-cell stage compared with normal embryos (Wang *et al.* 2024). The inhibition of Aurora A kinase (AURKA) is a cause of human embryo arrest (Wang *et al.* 2024). Its inhibition will inhibit the glycolysis process within the embryo and lead to the reduction in glucose metabolism level of fertilized eggs. At the same time, the microtubule cytoskeleton abnormality mediated by AURKA leads to embryo arrest in both humans and mice (Wang *et al.* 2024). Finally, many previous studies have shown that the regulation of glucose and lipid metabolism may control and determine the fate of the blastocyst (Bulut-Karslioglu *et al.* 2016, Tšuiko *et al.* 2019, Zhu *et al.* 2019, Hussein *et al.* 2020, Murphy 2020). For example, the inhibition of mechanistic target of rapamycin (mTOR) pathway can induce and maintain the development delay of cleavage stage embryos and the pause of blastocyst development (Bulut-Karslioglu *et al.* 2016, Zhu *et al.* 2019, Iyer *et al.* 2024), and its mechanism is closely related to glycolysis, pyruvate pathway, cholesterol metabolism and other related genes (Hussein *et al.* 2020).

There is evidence to indicate a major role of glucose in cellular differentiation, embryonic fate determination and organogenesis (Cao *et al.* 2024). Glucose metabolism operates through distinct pathways at critical stages of early embryogenesis: for example, the hexosamine biosynthetic pathway (HBP) modulates epiblast cell fate, and subsequently through glycolysis to facilitate mesodermal cell migration and tissue expansion. This two-wave metabolic activity highlights how glucose metabolism supports cell differentiation and migration across different developmental stages, providing a foundational understanding of metabolic regulation in early embryogenesis.

Through this review, we aim to provide a comprehensive understanding of the glucose metabolic underpinnings that support oocyte maturation and early embryonic development. We explore the roles of glycolysis, the pentose phosphate pathway (PPP) and the tricarboxylic acid cycle (TCA cycle), unraveling how these pathways not only fuel the developmental processes but also regulate critical events that dictate the successful progression from an oocyte to an embryo. Our focus on glucose metabolism is rooted in its ubiquitous presence and paramount importance in cellular processes, making it a cornerstone of developmental biology.

## Methods

The study is a narrative review. We searched the published articles in PubMed and Web of Science databases, containing words ‘Metabolism’, ‘Embryo metabolism’, ‘Oocyte metabolism’, ‘Glucose metabolism’, ‘Pyruvate metabolism’, ‘Embryo development’, ‘Oocyte maturation’, ‘Zygote genome activation’, ‘Maternal to zygotic genome’ and ‘Blastocyst differentiation’ in titles, abstracts or keywords. We selected articles published in English until November 2024. We used the EndNote reference manager to collect search results for initial assessment and exclusion of duplicate articles.

## Oocyte maturation and early embryonic development

Mammalian oocytes originate from primordial germ cells (PGCs), undergoing two stages: oocyte development and maturation. PGCs proliferate through mitosis during embryonic development, entering and arresting at the diplotene stage of prophase I, also known as the germinal vesicle (GV) stage. Hormonal cyclic changes trigger the resumption of meiosis, leading to organized GV breakdown (GVBD) and the expulsion of the first polar body (PB1) (Sen & Caiazza 2013). Concurrently, oocytes accumulate mRNA and proteins within the cytoplasm, while the cytoskeleton and organelles undergo rearrangement. Nuclear and cytoplasmic maturation together constitute oocyte maturation. During this process, surrounding cumulus cells expand, providing nutrients to the oocyte and engaging in bidirectional signaling to aid maturation. Mature oocytes then arrest again at metaphase II of meiosis, awaiting fertilization. Ovulated eggs are fertilized in the ampulla of the fallopian tube to form zygotes, initiating embryonic development (Li *et al.* 2013).

Early mammalian embryonic development is a complex process divided into two main stages, separated by the event of embryo implantation into the uterus. Preimplantation embryos spend most of their time freely floating in the reproductive tract, undergoing a series of cell divisions including two-cell, four-cell, eight-cell and morula stages. Initially, the zygotic genome is silent; thus, early embryonic development relies on substances accumulated within the oocyte. As cleavage proceeds, maternal mRNA and proteins are gradually cleared, leading to zygotic genome activation (ZGA), marked by an upsurge in genetic transcription – a process known as maternal-to-zygotic transition (MZT), one of the key events in embryonic development (Braude *et al.* 1988).

Subsequently, embryos undergo their first differentiation and form blastocysts with an internal cavity. Cells surrounding this cavity constitute the trophectoderm (TE), while those on one side of the cavity form the inner cell mass (ICM). The ICM further differentiates into epiblast and hypoblast, eventually developing into fetal structures. The TE envelops the ICM in human blastocysts and develops into embryonic outer layers that form placental structures (Ojosnegros *et al.* 2021). Thereafter, blastocysts hatch from the zona pellucida; a subgroup of TE cells interacts with uterine endometrial cells to implant (Fu *et al.* 2021).

## Basic glycolytic pathways in oocyte maturation and early embryonic development

Over the past decades, scientists have begun to unveil the significant contributions of metabolic states to oocyte maturation and preimplantation embryonic development. Metabolites not only provide the energy required for development, influencing the oxidative phosphorylation levels of gametes and embryos, but also act as triggers for critical events during development (Kaneko 2016).

For most organisms, glucose is the preferred carbon source for energy production (Dienel 2019, Zhao *et al.* 2023). Initially phosphorylated by hexokinase to glucose-6-phosphate, glucose enters glycolysis, yielding small amounts of ATP with the main chemical energy stored in pyruvate (Lunt & Vander Heiden 2011). Under anaerobic conditions, pyruvate often continues through glycolysis (Embden–Meyerhof–Parnas pathway) for a more rapid ATP production and turnover. With sufficient oxygen, pyruvate is transported into mitochondria, generating acetyl coenzyme A (acetyl-CoA) and entering the TCA cycle, resulting in complete substrate oxidation and more ATP production at the cost of generating reactive oxygen species (ROS) (Chakrabarty & Chandel 2021). In addition, glucose-6-phosphate can divert to PPP to produce nicotinamide adenine dinucleotide phosphate (NADPH), ribose-5-phosphate (R5P) and xylulose-5-phosphate (X5P), with the latter two being important biosynthetic precursors in cell proliferation (Tian *et al.* 1998, Kroemer & Pouyssegur 2008, Cairns *et al.* 2011). Glucose-6-phosphate can also be metabolized through the HBP, synthesizing UDP-N-acetylglucosamine (Ai *et al.* 2023). UDP-N-acetylglucosamine is then used for posttranslational modification (O-linked β-N-acetylglucosamine modification) of intracellular proteins involved in nutrient sensing and stress response regulation (Ferrer *et al.* 2014). It is responsible for protein glycosylation, a common posttranslational modification of membrane components.

During oocyte maturation and early embryonic development, glucose and pyruvate metabolism exhibit both conservation and differences among mammalian species. Similar to humans, mouse oocytes rely on granulosa cells to metabolize glucose into pyruvate, which is then transported via gap junctions to provide energy for the oocyte (Roberts *et al.* 2004, Gu *et al.* 2015). When glycolysis is inhibited, mouse oocytes can initiate alternative metabolic pathways to sustain maturation (Paczkowski *et al.* 2013). However, human oocytes seem to have higher glycolytic enzyme activity (Chi *et al.* 1988). In both species, glucose consumption significantly increases after the eight-cell/morula stage, marking a shift toward aerobic glycolysis that supports blastocyst formation (Gardner & Leese 1986, Leese *et al.* 1993). In bovine, exogenous glucose is essential for oocyte maturation, although glucose and pyruvate uptake remain low in early embryos until the 16-cell stage. Glucose demand then increases significantly as the embryo progresses to the blastocyst stage (de Souza *et al.* 2015, Gu *et al.* 2015). By contrast, porcine oocytes have abundant endogenous glucose and triglyceride reserves, with a marked increase in glycolytic demand at the blastocyst stage, indicating a growing reliance on glucose metabolism as development advances (Sturmey & Leese 2003, Paczkowski *et al.* 2013).

Overall, although the balance of energy sources varies across species, glucose and pyruvate metabolism universally serve as core energy sources, peaking in demand at the blastocyst stage. This review focuses on the key aspects of glucose metabolism in mice and humans during this process.

## Metabolic regulatory networks between glycolysis and fatty acid, amino acid and nucleotide metabolism in early-life processes

Apart from glucose and pyruvate metabolism, fatty acids (FAs), amino acids and nucleotides play key roles in gamete and embryo development, interrelating with glucose metabolism. Fatty acid oxidation (FAO) supports ATP and NADPH synthesis, crucial for energy, oxidative stress regulation and signal modulation in oocytes and early embryos. During oocyte maturation, FAO – facilitated by enzymes such as carnitine palmitoyltransferase 1B – promotes meiosis and developmental competence, while polyunsaturated fatty acids (PUFAs), such as arachidonic acid, docosahexaenoic acid and eicosapentaenoic acid, help regulate meiotic resumption, although excess PUFAs can disrupt chromosomal alignment (Dunning *et al.* 2010, Li *et al.* 2020*b*, de Lima *et al.* 2023).

In early embryonic development, β-oxidation genes are upregulated from cleavage to the blastocyst stage, supporting β-oxidation as a vital metabolic pathway. Increased unsaturated FAs at the blastocyst stage enhance membrane fluidity, morphology and antioxidative protection (Sharpley *et al.* 2021, Zhang *et al.* 2024). Interestingly, glucose and lipid metabolism appear to be interconnected rather than merely parallel processes. At the two-cell stage, glucose supplementation significantly upregulates genes related to lipid metabolism, including those involved in fatty acid, ceramide and long-chain acyl-CoA synthesis. This suggests that glucose not only serves as an energy source but also enhances lipid metabolism to support oocyte maturation and early embryonic development (Wang *et al.* 2023*b*).

Amino acids play essential roles in early embryonic development by acting as nitrogen sources and regulating energy metabolism. Normal embryos show specific amino acid profiles, such as asparagine, glutamine and arginine, which differ from arrested embryos (Houghton *et al.* 2002, Hong *et al.* 2023). Lane & Gardner found that mouse blastocysts cultured without amino acids and vitamins had increased glycolysis and reduced oxidative capacity, lowering developmental potential; supplementation restores oxidative balance and viability comparable to *in vivo* levels (Lane & Gardner 1998). The malate–aspartate shuttle (MAS) transfers NADH from the cytosol to mitochondria, facilitating carbohydrate utilization for ATP from the two-cell stage onward. MAS activity increases from the two-cell to the eight-cell stage, enabling efficient glucose and lactate utilization (Faff-Michalak & Albrecht 1991, Lane & Gardner 2005). Inhibition of MAS reduces glucose uptake and impairs oxidative capacity, impacting growth, differentiation and implantation (Mitchell *et al.* 2009). Branched-chain amino acids (BCAAs) such as leucine enhance glucose uptake and consumption and may support blastocyst development via the mTOR pathway (Doi *et al.* 2003, Gangloff *et al.* 2004, Zhang *et al.* 2017*a*). Thus, amino acids are crucial not only as energy sources but also as regulators of glucose metabolism, affecting embryonic quality and development.

During early embryonic development, glucose metabolism not only supplies energy but also provides essential precursors for nucleotide synthesis. Leese and coworkers first proposed the importance of nucleotides in the regulation of embryonic metabolism in 1984 (Leese *et al.* 1984). Recent studies indicate that in the early stages of development (e.g. the two-cell stage), nucleotide synthesis primarily depends on maternal nucleotide reserves (Chi *et al.* 2020). As the embryo progresses to the morula stage, the demand for nucleotide synthesis increases (Chi *et al.* 2020). Glucose contributes to this process through PPP and, along with sphingosine-1-phosphate (S1P) signaling, enables the synthesis of glucose-dependent nucleotides (Chi *et al.* 2020). Meanwhile, pyruvate and endogenous metabolites contribute to base formation, resulting in new nucleotide synthesis (Sharpley *et al.* 2021). This process indicates that following ZGA, the cooperative roles of glucose and pyruvate in nucleotide synthesis support the proliferation and differentiation of early embryos, providing essential substrates and energy for subsequent development.

## Key glucose metabolism during oocyte maturation

**Figure 1:**
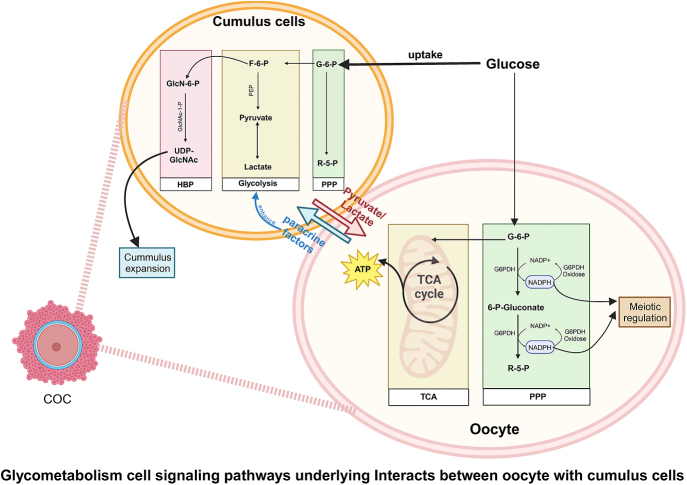
A diagram of mechanisms of glucose metabolism within the cumulus–oocyte complex. The carbohydrate metabolism in oocytes and cumulus cells is distinct yet closely related. Cumulus cells collect glucose from follicular fluid and convert it into pyruvate through glycolysis. Glucose in cumulus cells can also be metabolized via the pentose phosphate pathway (PPP) and hexosamine biosynthesis pathway (HBP). The products of the HBP are converted into hyaluronic acid, which aids cumulus cells in synthesizing the extracellular matrix, facilitating cumulus expansion. Oocytes primarily utilize pyruvate provided by cumulus cells for the tricarboxylic acid (TCA) cycle and oxidative phosphorylation to generate energy. In addition, oocytes regulate glycolysis within cumulus cells through paracrine signaling factors. A small portion of glucose can be metabolized via the PPP pathway, producing nicotinamide adenine dinucleotide phosphate (NADPH), which helps clear reactive oxygen species generated during oxidative phosphorylation, thus assisting in meiosis and nuclear maturation of oocytes. COC, cumulus–oocyte complex; HBP, hexosamine biosynthetic pathway; PPP, pentose phosphate pathway; TCA, tricarboxylic acid cycle; ATP, adenosine triphosphate; G-6-P, glucose-6-phosphate; F-6-P, fructose-6-phosphate; R-5-P, ribose-5-phosphate; GlcN-6-P, glucosamine-6-phosphate; GlcNAc-1-P, N-acetylglucosamine 1-phosphate; UDP-GlcNAc, uridine diphosphate N-acetylglucosamine; PEP, phosphoenolpyruvate; G6PDH, glucose-6-phosphate dehydrogenase; NADP+, nicotinamide adenine dinucleotide phosphate (oxidized form); NADPH, nicotinamide adenine dinucleotide phosphate (reduced form).

The environment to which cumulus–oocyte complexes (COCs) are exposed during *in vivo* or *in vitro* maturation (IVM) profoundly influences fertilization and subsequent embryonic development (Richani *et al.* 2024). Glucose, a pivotal metabolite for COCs, is metabolized through glycolysis, the PPP and the HBP (Fig. 1).

Oocytes themselves have a significantly lower capacity for glucose absorption and metabolism compared to cumulus cells, due to differences in glucose transporter types and affinities (Roberts *et al.* 2004) and disparities in the activities of phosphofructokinase and glucose-6-phosphate dehydrogenase (Cetica *et al.* 2002). Consequently, cumulus cells harvest glucose from follicular fluid, converting it via glycolysis into pyruvate and lactate to supply oocytes. In COCs at the end of IVM, glucose flux through the HBP pathway significantly increases (Sutton-McDowall *et al.* 2004). Most produced UDP-N-acetylglucosamine is converted into hyaluronic acid to aid cumulus cells in extracellular matrix (ECM) synthesis for cumulus expansion (Gutnisky *et al.* 2007). Concurrently, the HBP pathway also facilitates O-linked glycosylation of proteins, altering their conformation and subsequently activating downstream targets, leading to upregulation or downregulation of protein signaling pathways (Wells *et al.* 2003) (Fig. 1).

Within oocytes, these substrates primarily fuel the TCA and oxidative phosphorylation for energy production, with a minor portion participating in glycolysis (Dan-Goor *et al.* 1997) (Fig. 1). However, oxidative phosphorylation inevitably leads to ROS production, which is counterbalanced by intracellular antioxidants (von Mengden *et al.* 2020). Notably, despite lower glycolytic activity in oocytes, studies indicate a significant positive correlation between this reduced activity and oocyte developmental competence (Herrick *et al.* 2006). In addition, oocytes upregulate glycolytic enzyme genes (*Pfkp* and *Ldha*) in cumulus cells via paracrine factors to ensure adequate pyruvate delivery (Sugiura *et al.* 2005) (Fig. 1). Metabolic dynamics studies of *in vivo* oocytes at GV stage, GVBD stage and MII stage reveal (Li *et al.* 2020*b*) that key TCA cycle components – citrate, cis-aconitate and fumarate – exhibit varying degrees of increase during maturation. Correspondingly, most TCA cycle enzymes (8 out of 10) also show a consistent rise. In harmony with this, the constituent enzymes of the pyruvate dehydrogenase (PDH) complex also increase during oocyte maturation.

Beyond energy provision, oocytes also activate the PPP (Fig. 1). In oocytes, PPP activity accounts for a minor portion of mouse oocyte glucose metabolism (<3%) (Urner & Sakkas 1999), yet it impacts meiotic division (Downs *et al.* 1998, Kang *et al.* 2021), postfertilization cleavage and blastocyst formation (Herrick *et al.* 2006, Gutnisky *et al.* 2014). Significant fluctuations are observed in PPP’s two primary initial metabolites and several key intermediates during oocyte maturation (Li *et al.* 2020*b*). Moreover, five out of six PPP-related enzymes exhibit statistically significant increase during meiotic maturation (Li *et al.* 2020*b*). siRNA-mediated downregulation of glucose-6-phosphate dehydrogenase (G6PD), the rate-limiting enzyme of PPP, revealed that reducing G6PD (siG6pdx) in oocytes had a slight but significantly reduced maturation rate. Furthermore, mature siG6pdx oocytes exhibit a decreased proportion of two-cell embryo formation following *in vitro* fertilization (IVF), indicating an impaired transition from an oocyte to an embryo (Li *et al.* 2020*b*). A major phenotype of siG6pdx oocytes is excessive ROS production, suggesting PPP’s role in redox balance regulation (Li *et al.* 2020*b*). A PCOS mouse model established through high-androgen levels indicates that DHEA-supplemented mouse oocytes exhibit markedly lower levels of citrate, G6PDH activity and lipid content compared to controls suggesting TCA and PPP metabolic abnormalities (Jimenez *et al.* 2013). Women with PCOS and hyperandrogenism typically feature increased numbers of retrieved oocytes during IVF. However, these oocytes are often of poor quality, resulting in reduced fertilization/implantation rates and increased miscarriage rates (Qiao & Feng 2011).

Maternal glucose metabolism environment is also influenced by gametes, further affecting offspring through epigenetic mechanisms. TET3 is the sole DNA 5-methylcytosine (5 mC) modifying enzyme expressed during oocyte development. It catalyzes the conversion of 5 mC to 5-hydroxymethylcytosine (5hmC) and other highly oxidized products, playing a crucial role in DNA demethylation during the zygotic stage (Wossidlo *et al.* 2011). Studies in diabetic patients, hyperglycemic rats and mouse models indicate (Chen *et al.* 2022) that TET3 expression in MII oocytes significantly decreases with increasing glucose concentration in follicular fluid, ultimately resulting in insufficient DNA demethylation at the pronuclear stage 3–4 (PN3–4) zygotes derived from IVF. Impaired DNA demethylation and epigenetic changes can affect the expression of a subset of paternally hypermethylated genes involved in insulin secretion, including glucokinase gene (*Gck*), which is essential for glucose metabolism, thereby rendering offspring sensitive to glucose intolerance.

## Pyruvate regulates ZGA

Early embryos float within the fallopian tubes and uterus, developing in these environments. Human early embryos have limited internal energy stores; thus, the reproductive tract fluids provide essential nutrients and metabolic substrates, primarily pyruvate, lactate, glucose and amino acids. These nutrients have independent and overlapping metabolic functions (Bavister 1995, Gardner *et al.* 1996). Before differentiation in mouse and human embryos during the cleavage stage, pyruvate appears to be the central energy substrate for energy demands (Gardner & Leese 1986, Hardy *et al.* 1989), regulating embryonic genome activation.

There is a critical window for pyruvate during early embryonic development, mapped to the 30 h between 24 and 54 h of mouse embryo development (Nagaraj *et al.* 2017). Deprivation of pyruvate during this window leads to developmental arrest at the two-cell stage, accompanied by a sharp increase in NADH levels and delayed expression of metabolic genes (, Nagaraj *et al.* 2017, Sharpley *et al.* 2021). Transcriptome analysis indicates that at the two-cell stage, zygotic cells downregulate genes related to glucose uptake and glycolysis while upregulating those associated with mitochondrial pyruvate metabolism and oxidative phosphorylation. The expression of peroxiredoxin genes *Prdx1* and *Prdx2* decreases (Sun *et al.* 2023). This critical window for pyruvate requirement aligns astonishingly with the MZT timeline in both mice and humans (Zou *et al.* 2022), providing key evidence that pyruvates are indispensable for MZT. MZT involves maternal mRNA clearance and timely ZGA, suggesting that pyruvate may facilitate the clearance of maternal mRNAs and activation of the zygotic genome.

**Figure 2:**
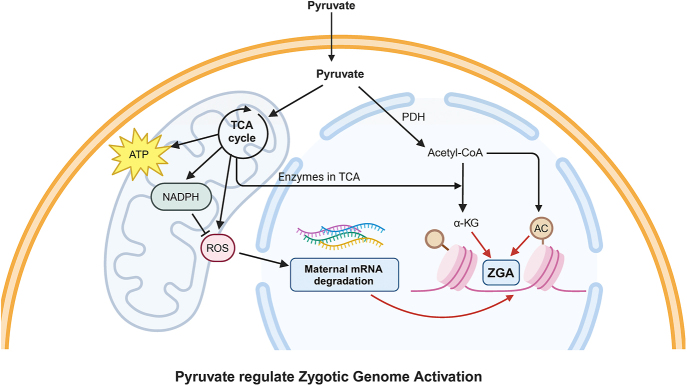
A diagram of mechanisms of pyruvate regulating zygote genome activation in the early embryo. During the cleavage stage of the embryo, zygotic genome activation relies on pyruvate. On the one hand, pyruvate influences the selective translocation of key mitochondrial tricarboxylic acid cycle proteins to the nucleus, thereby affecting histone acetylation and chromatin remodeling. On the other hand, pyruvate may assist in clearing maternal messenger ribonucleic acids by generating nicotinamide adenine dinucleotide phosphate (NADPH) and reducing reactive oxygen species (ROS). mRNA, messenger ribonucleic acid; PDH, pyruvate dehydrogenase; acetyl-CoA, acetyl coenzyme A; ROS, reactive oxygen species; AC, acetyl; α-KG, alpha-ketoglutarate; TCA, tricarboxylic acid cycle; ATP, adenosine triphosphate; NADPH, nicotinamide adenine dinucleotide phosphate (reduced form).

On the one hand, at the end of the two-cell stage in mouse embryos, as mentioned earlier, most maternal RNAs and some proteins are depleted, necessitating embryonic genome activation for further development (Li *et al.* 2020*a*). This significant genomic reprogramming requires metabolic products dependent on mitochondrial enzymes driving the TCA cycle and the utilization of pyruvate by PDH. In addition, studies suggest that pyruvates may assist in clearing maternal mRNAs by promoting m- and z-decay pathways, possibly by disrupting them, thus enabling early mouse embryos to smoothly transition beyond the two-cell stage (Fig. 2).

On the other hand, during the critical ZGA period, not only an increased energy is required, but also requires structural changes and epigenetic remodeling. These processes are essential for the transition from the maternal/paternal genome to the embryonic genome. This transition relies on the epigenetic remodeling of mitochondrial enzymes associated with the TCA cycle (Fig. 2). Pyruvates are essential for these mitochondrial enzymes’ nuclear localization during ZGA (Nagaraj *et al.* 2017). Zygote-derived pyruvate dehydrogenase α1 (*PDHAl*) expression promotes porcine ZGA by maintaining histone acetylation levels in pig embryos (Zhou *et al.* 2020). Acetyl-CoA generated from pyruvate can serve as a cofactor for histone acetyltransferases (HATs) (Martínez-Reyes & Chandel 2020). This means that pyruvate both influences selective translocation of the key mitochondrial TCA cycle proteins to the nucleus and directly or indirectly affects histone acetylation and chromatin remodeling (Zhang *et al.* 2018, Zhou *et al.* 2021).

Furthermore, pyruvates impact early embryonic development by reducing oxidative stress levels (Zhang *et al.* 2022). The low metabolic activity at early stages is thought to maintain low ROS levels, thereby avoiding DNA damage during the totipotency phase (Karja *et al.* 2006, Baumann *et al.* 2007).

## Glucose assists in determining the fate of the blastocyst

Glucose is a crucial metabolic substance in the fallopian tube fluid, essential for preimplantation embryonic development (Devreker *et al.* 2000). During blastocyst formation, glucose is preferentially converted to lactate through aerobic glycolysis, even under ample oxygen conditions. This phenomenon is known as the Warburg effect, first described by Warburg in 1956 in cancer cells (Warburg 1956). Similar to cancer cells, blastocysts exhibit preferential glycolysis, highlighting its essential role during blastocyst formation and implantation (Gardner & Leese 1990, Harris *et al.* 2005).

During early cleavage stages, both mice and humans cannot metabolize glucose alone. Compared to pyruvate, glucose metabolism appears to generate more ROS, potentially damaging the embryo’s genetic material (Karja *et al.* 2006, Baumann *et al.* 2007, Zhang *et al.* 2022). In addition, studies have shown that high concentrations of citrate in cleavage-stage cells inhibit the key enzyme phosphofructokinase-6, which is also associated with inhibited glucokinase activity (Barbehenn *et al.* 1974, Leese *et al.* 1993). However, pyruvate and lactate can only support embryonic development up to the morula stage. Their uptake rates gradually decrease from the eight-cell stage onward, while glucose becomes increasingly essential for blastocyst formation (Brown & Whittingham 1991, Lane & Gardner 2000, Donnay *et al.* 2002). Specifically, the critical window for glucose uptake ranges from 76 to 82 h post-hCG injection in mice (Brown & Whittingham 1991). Providing ample glucose beyond this window does not increase blastocyst formation rates (Martin & Leese 1999), indicating that there is a specific period during which glucose availability is crucial for development.

The reasons behind these changes in embryonic glucose requirements during development are still under investigation. At the morula stage, metabolic activity (measured by oxygen consumption) surges across all studied species, primarily due to energy demands for sodium pumping during blastocoel formation and increased protein synthesis as embryos begin net growth (Sturmey *et al.* 2009). Key glycolytic enzymes such as glucose phosphate isomerase, triose phosphate isomerase and glyceraldehyde 3-phosphate dehydrogenase are consistently upregulated from the zygote to blastocyst stage in both mice and humans (Li *et al.* 2022, Malkowska *et al.* 2022), and mutations in these enzymes have been shown to cause embryonic lethality (Merkle & Pretsch 1989, West *et al.* 1990, Pretsch 2000). Correspondingly, this metabolic shift and increased energy demand are accompanied by changes and proliferation in glucose transporter proteins (Augustin *et al.* 2001, Arhin *et al.* 2019), particularly glucose transporter 1, 3 and 8 (GLUT 1, 3 and 8). Targeted degradation or knockout of each GLUT leads to adverse outcomes (Purcell & Moley 2009). Apoptosis induced in both model systems correlates with reduced expression of GLUT1 within the plasma membrane (Riley *et al.* 2006).

**Figure 3:**
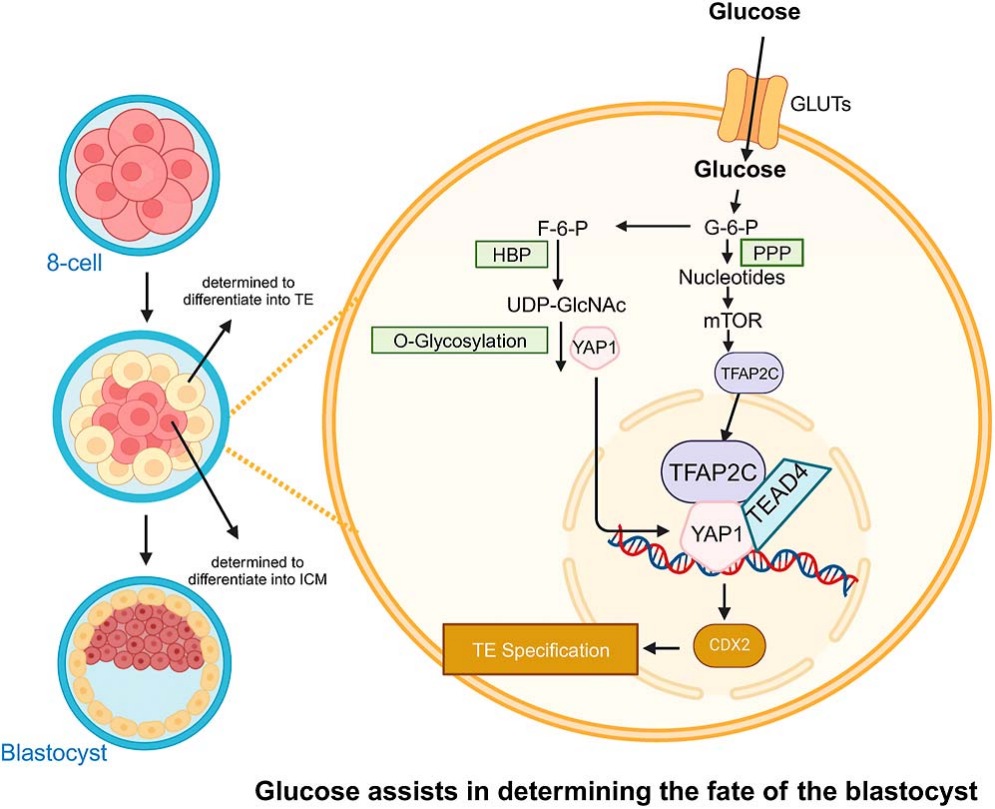
A diagram of mechanisms of glucose controlling the fate of blastocyst. During the transition from the eight-cell stage to blastocyst formation, glucose serves as a signaling transducer in cell metabolism. Glucose, via the hexosamine biosynthesis pathway (HBP), leads to protein O-GlcNAcylation of YAP1, directing it to the cell nucleus. In addition, through the pentose phosphate pathway (PPP), glucose-dependent nucleotide synthesis activates mTOR and enables Tfap2c translation. The interaction between YAP1, TEAD4 and TFAP2C forms a complex that activates CDX2 expression, controlling transcription of TE specific genes. O-GlcNAcylation, O-linked N-acetylglucosamine glycosylation; YAP1, yes-associated protein; mTOR, mechanistic target of rapamycin; TFAP2C, transcription factor AP-2 gamma; TEAD4, TEA-domain family member 4; CDX2, caudal type homeobox 2; GLUTs, glucose transporters; HBP, hexosamine biosynthetic pathway; PPP, pentose phosphate pathway; G-6-P, glucose-6-phosphate; F-6-P, fructose-6-phosphate; UDP-GlcNAc, uridine diphosphate N-acetylglucosamine; TE, trophectoderm; ICM, inner cell mass.

Glucose seems to be more than just a metabolic substrate; it also acts as a cell signaling agent, influencing the differentiation of the blastocyst. Through carbon labeling and tracking in glucose, researchers have found that metabolites from the TCA cycle do not carry glucose carbon labels (Chi *et al.* 2020). In other words, glucose’s primary role in embryos appears not to be providing energy for ATP formation in mitochondria; energy still comes from pyruvate and lactate. Glucose controls TE cell fate programmatically through these metabolic processes (Fig. 3). The determination of trophectoderm lineage fate requires expression of the key trophectoderm lineage indicator factor *Cdx2*, a prerequisite for blastocyst formation. Early studies indicate that *Cdx2* expression is regulated by transcription factors including *Tfap2c*, *Tead4/YAP* complex and Notch pathway (Nishioka *et al.* 2009, Rayon *et al.* 2014, Cao *et al.* 2015); YAP’s binding partner *Tead4* can function within mitochondria to regulate trophectoderm differentiation (Kaneko & DePamphilis 2013). Glucose-dependent nucleotide synthesis via the PPP and S1P signaling activates mTOR, allowing translation of *Tfap2c*. Interactions between YAP1, TEAD4 and TFAP2C form a complex controlling TE-specific gene transcription (Chi *et al.* 2020).

Another important aspect of the increased glucose demand during blastocyst formation is its role in metabolic flexibility. This preference for aerobic glycolysis not only supports a rapid supply of ATP but also influences the local environment critical for implantation by producing lactate. In mouse blastocysts, 55% of glucose consumed by the TE and 100% by the ICM can be accounted for by lactate formation (Hewitson & Leese 1993). Similarly, in human blastocysts, approximately 90% of lactate production is attributable to exogenous glucose metabolism (Gott *et al.* 1990).

The accumulation of lactate significantly influences the implantation process by modulating various cell types and the ECM (Gardner 2015, Ma *et al.* 2020, Gurner *et al.* 2022). Specifically, lactate promotes the activation of matrix metalloproteinases and cathepsin B, induces hyaluronan synthesis and suppresses excessive proliferation of endometrial cells. These processes lead to ECM degradation, reduced tight junctions between cells, increased extracellular space and enhanced trophoblast invasion. In addition, lactate acts on macrophages and endothelial cells, promoting the expression and release of vascular endothelial growth factor, which stimulates angiogenesis at the maternal–fetal interface. Lactate also exerts immunomodulatory effects by reducing the cytotoxicity of T cells and natural killer cells, promoting Th2-type immune responses and helping regulate the local immune environment to ensure that the embryo is protected from maternal immune system attacks, ultimately increasing the success rates of implantation (Gardner 2015, Ma *et al.* 2020).

## Future perspectives in embryo selection and personalized ART: maternal metabolism, noninvasive monitoring and epigenetic insights

### Maternal metabolism and early nutritional exposure: implications for offspring metabolic health

Numerous studies have confirmed that maternal metabolism and early nutritional exposure significantly impact the metabolic health of offspring through metabolic programming during fetal development, thereby increasing the risk of metabolic diseases in adulthood (Wankhade *et al.* 2016, 2018, Norris *et al.* 2017, de Sousa *et al.* 2018). Therefore, attention to maternal metabolism should begin at the earliest stages of life, spanning oocyte development, early embryo stages and postimplantation. Excess glucose in the embryonic environment originates from maternal glucose metabolism disorders. Although the embryo does not directly depend on blood circulation, its metabolic state is closely linked to maternal conditions. The glucose in follicular fluid, for instance, is derived from plasma (Gardner & Leese1990, Leroy *et al.* 2004), and metabolites found in the blood of diabetic mothers can transfer to follicular fluid via vascular cells. In mice, glucose concentrations in oviductal fluid are similar to those in serum (Schindler *et al.* 2020). In addition, the metabolites in embryonic fluids are closely related to those in maternal blood (Hadi *et al.* 2005, Gürke *et al.* 2015). Maternal metabolic disorders, such as insulin resistance or insulin deficiency, can compromise oocyte and embryo quality, leading to lower pregnancy rates. Studies have shown that women with PCOS and premature ovarian insufficiency exhibit dysfunctional glycolysis and pyruvate metabolism in follicular fluid, reducing the success rate of assisted reproductive technology (ART) (Wang & Wu 2020, Stener-Victorin *et al.* 2024).

Currently, many studies have reported positive outcomes from nutritional and metabolic interventions during pregnancy and lactation (Dodd *et al.* 2010, Teede *et al.* 2022, Martínez-Vizcaíno *et al.* 2023). However, a significant proportion of studies on maternal metabolic interventions show limited effectiveness in improving maternal and neonatal health outcomes (Ronnberg *et al.* 2017, Grotenfelt *et al.* 2020). A recent study with extensive data summarizes the complex effects of maternal obesity on pregnancy outcomes and offspring health, especially concerning pregnancy complications and the long-term metabolic and neurological health of the offspring. The study suggests that weight management interventions, such as dietary control and exercise, implemented before or during pregnancy have shown limited efficacy in improving offspring body fat and weight indices (Yang *et al.* 2024).

Further research is needed in this field to explore how improving maternal metabolic status, particularly by regulating glucose intake and enhancing insulin sensitivity, can improve oocyte quality and embryo development potential. This could enable the inclusion of metabolic intervention strategies in personalized ART protocols to enhance pregnancy success rates.

### Glucose metabolism, epigenetic regulation and insights from cancer research in embryo development

Glucose metabolism and its enzymatic products play critical regulatory roles in embryo development through various epigenetic mechanisms. In mouse embryos, distinct metabolic signatures are observed between the two-cell and blastocyst stages. For example, levels of 2-hydroxyglutarate (2-HG) are elevated in two-cell embryos, while citrate and α-ketoglutarate (α-KG) are more abundant in blastocysts. *In vitro* studies show that adding L-2-HG delays embryo development and increases H3K4 trimethylation, thereby reducing blastocyst quality (Zhao *et al.* 2021). A key enzyme in glycolysis, enolase 1 (ENO1), also contributes to metabolic reprogramming in early embryos under the influence of RNA-binding proteins, which is essential for cell differentiation (Huppertz *et al.* 2022).

Both cancer cells and early embryonic cells exhibit high glycolytic metabolism (Warburg effect), a model that supports rapid proliferation and supplies key metabolites for epigenetic modifications. Cancer research indicates that glucose can induce NEDD4-mediated ubiquitination and subsequent acetylation of histone H3, regulating the expression of tumor-related genes and promoting cancer cell proliferation, highlighting the close relationship between high-glucose metabolism and epigenetic regulation (Zhang *et al.* 2017*b*, You *et al.* 2023). During early embryo development, glucose metabolites similarly modulate gene expression through acetylation pathways. For example, acetyl-CoA facilitates SMAD3 acetylation, promoting the differentiation of embryonic stem cells into the endoderm, while NAD+ works in tandem with *Sirt1* to mediate H3K27ac deacetylation, precisely regulating gene expression during ZGA (Li *et al.* 2022, Yi *et al.* 2023). NEDL2 (Nedd4-related E3 ubiquitin ligase-2), a member of the NEDD4 family, may play a similar role in epigenetic regulation during embryo development, particularly in chromatin structure modulation and gene expression (Mao *et al.* 2021).

Another key epigenetic pathway is O-linked β-N-acetylglucosamine (O-GlcNAc) modification, which is widely present in the cytoplasm and nucleus, influencing nutrient sensing, cell cycle regulation and transcription (Torres & Hart 1984, Roos *et al.* 1997, Hart *et al.* 2007). In hepatocellular carcinoma cells, elevated glucose levels increase O-GlcNAc concentration, promoting cell proliferation and activating the eIF4E axis to maintain stem cell-like potential (Zeidan *et al.* 2010). Similarly, O-GlcNAc glycosylation plays an essential role in maintaining ESC pluripotency, with higher O-GlcNAc levels supporting the activation of key transcription factors such as *Oct4*, *Sox2* and *Nanog* (Jang *et al.* 2012, Myers *et al.* 2016, Hao *et al.* 2019).

In conclusion, glucose metabolism and its metabolites play essential roles in embryonic cell differentiation and fate determination through various epigenetic pathways, such as methylation, acetylation and O-GlcNAc glycosylation. This mechanism parallels findings in cancer research and provides insights into exploring epigenetic regulation in embryo development.

### Prospects for noninvasive metabolic monitoring in embryo selection and personalized assisted reproductive therapy

Selecting embryos with high reproductive potential is essential for enhancing the success rates of ART. However, traditional morphological assessment methods fail to capture the full spectrum of embryo health and developmental potential (Kupka *et al.* 2016, Cutting 2018). With the recent trend toward single embryo transfers, selecting embryos with higher developmental potential, particularly blastocysts, has become critical to improving ART outcomes (Zeng *et al.* 2018, Li *et al.* 2021). In this context, metabolomics has shown significant promise. For instance, reduced β-oxidation, increased glycolysis and a markedly decreased ATP/ADP ratio in IVM oocytes all suggest that early embryonic metabolic states closely correlate with developmental potential (Frolova *et al.* 2011, Herta *et al.* 2022).

To minimize potential harm from traditional biopsy techniques, research is increasingly focusing on noninvasive metabolic monitoring. Seli and coworkers used near-infrared spectroscopy to detect differences in metabolite concentrations in embryo culture media, deriving an ‘embryo viability score’ that correlates significantly with implantation and pregnancy success rates (Seli *et al.* 2007, 2010, Scott *et al.* 2008). Other studies have demonstrated that blastocysts with similar morphological grades can still exhibit distinct proteomic profiles and glucose uptake levels, further validating the value of metabolomics in predicting embryo quality (Gardner *et al.* 2001, Katz-Jaffe *et al.* 2006). Large-scale analyses of embryo culture media samples have confirmed the utility of the ‘embryo viability score’ in predicting implantation rates and fetal cardiac activity (Seli *et al.* 2011).

Specifically within glucose metabolism, blastocysts with higher Gardner scores, KIDScores or EmbryoScores consume at least 40% more glucose, which is associated with higher developmental potential and pregnancy success rates (Ferrick *et al.* 2020). In addition, NMR spectroscopy of follicular fluid in PCOS patients shows a positive correlation between lower glucose uptake and embryo development, supporting adjustments to glucose levels in *in vitro* culture (Castiglione Morelli *et al.* 2019). Other metabolic markers, such as pyruvate, succinate and malate in PCOS follicular fluid, serve as important indicators of embryo quality (Zhao *et al.* 2015). Recent studies also indicate that glycan patterns in spent culture media may provide noninvasive clues regarding embryo development and embryo–uterine interactions (Wang *et al.* 2023*a*).

With advancements in noninvasive imaging, fluorescence lifetime imaging microscopy (FLIM) offers a sensitive means to detect subtle metabolic differences in oocytes and early embryos by measuring fluorescence lifetime data from NAD(P)H and FAD+, highlighting metabolic states (Tan *et al.* 2022, Venturas *et al.* 2022, 2023). FLIM also detects metabolic variations across embryo regions, such as the ICM and TE (Shah *et al.* 2022, Sakkas *et al.* 2024), revealing potential applications in ploidy assessment, although further validation for pregnancy outcome prediction is needed.

Moreover, the combination of two-photon autofluorescence imaging and AI analysis has revealed significant fluorescence phase differences in embryos under varying metabolic conditions, particularly for glucose, pyruvate and lactate levels, suggesting new possibilities for personalized embryo selection (Parra *et al.* 2024). Time-lapse systems integrated with artificial intelligence provide more comprehensive embryo monitoring and supporting analyses such as morphological grading, blastocyst formation, aneuploidy prediction and live birth prediction (Luong & Le 2024).

Overall, integrating metabolomics, noninvasive imaging and AI-based analysis offers a promising approach for the precise selection of high-quality gametes and embryos. Although these technologies still require further validation to ensure that they have no potential adverse effects on embryo development, they are likely to play a key role in personalized ART treatments in the future.

## Conclusion

**Figure 4:**
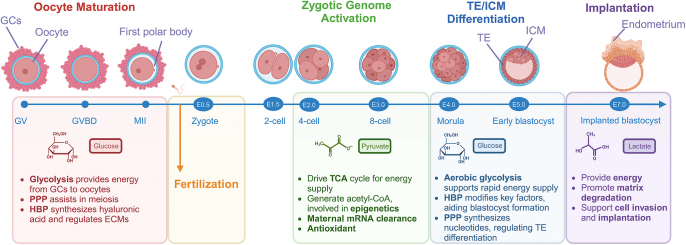
Key glucose metabolic pathways and the roles of metabolites during human development from gametes to embryo implantation. Oocyte maturation, fertilization, zygotic genome activation, blastocyst differentiation and implantation are key events in early human development. This figure illustrates the important metabolic processes involving glucose, pyruvate and lactate, and their unique roles in these critical events.

Glucose metabolism plays a pivotal role in oocyte maturation and early embryonic development (Fig. 4). Traditionally, metabolism is considered to have two main functions: providing the energy required by cells to maintain intracellular homeostasis and support specific functions and supplying metabolites for the biosynthesis and export of cellular components. Recent studies summarized in this article suggest that metabolism also supports tissue development by providing metabolic intermediates for energy production, synthetic metabolism, epigenetic regulation of gene expression and formation of metabolic gradients (Solmonson *et al.* 2022).

Some of the most intriguing events occur during the beginning of mammalian life, such as oocyte maturation, clearance of maternal mRNAs, activation of the zygotic genome and formation and differentiation of blastocysts. Oocyte maturation and early embryo development depend on exogenous glucose metabolites in the follicular fluid and reproductive ducts, which not only provide energy for embryonic development but also play a regulatory and signaling role at critical moments of embryonic development. Pyruvate plays a key role in early embryonic development. Exogenous pyruvate promotes ZGA and further development of early embryos by influencing the localization of mitochondrial enzymes (Sharpley *et al.* 2021), metabolic flux in the TCA cycle (Sharpley *et al.* 2021), clearance of maternal mRNAs (Zhang *et al.* 2021) and reducing oxidative stress levels (Zhang *et al.* 2022). Glucose has multiple roles in oocyte maturation and preimplantation embryo development. On the one hand, it serves as a metabolic substrate supporting oocyte maturation and blastocyst formation (Devreker *et al.* 2000). On the other hand, it is involved in biosynthesis, cell signaling, epigenetic remodeling and determination of blastocyst cell fate (Chi *et al.* 2020).

This review explores the central roles of glucose and pyruvate metabolism in oocyte maturation and early embryonic development, highlighting the indispensable influence of metabolism in these processes. As research progresses, future science will increasingly focus on optimizing maternal metabolic environments, exploring the complex interactions between metabolism and epigenetics and utilizing noninvasive monitoring technologies, such as metabolomics and imaging analysis, to precisely assess embryonic development potential. These advancements will not only offer new therapeutic strategies for personalized ART but also usher in a new era of precision medicine, providing unprecedented hope and possibilities for families facing fertility challenges.

## Declaration of interest

The authors declare that there is no conflict of interest that could be perceived as prejudicing the impartiality of the research reported.

## Funding

This work was supported by the National Science Fund for Distinguished Young Scholarshttps://doi.org/10.13039/501100014219 (82225019 to YY), the National Key Research Development Chinahttps://doi.org/10.13039/501100012166 and Program (2021YFC2700303 to YY), the National Natural Science Foundation of Chinahttps://doi.org/10.13039/501100001809 (82192873, 81971381, 82101714 and 81771580 to YY), the Beijing Nova Programhttps://doi.org/10.13039/501100005090 (20240484604 to YY) and the China Postdoctoral Science Foundationhttps://doi.org/10.13039/501100002858 (2024M750654 to YBW).

## Author contribution statement

Y Zhang, Y Wang and Y Yu are responsible for establishing the review idea, gathering information and writing. T Li participated in the discussion of the perspective. All authors read and approved the final manuscript.

## Data availability

All data are available via the corresponding authors.
